# Optimizing the dynamic zero-COVID policy in China

**DOI:** 10.7150/ijbs.75699

**Published:** 2022-08-21

**Authors:** Wei Bai, Sha Sha, Teris Cheung, Zhaohui Su, Todd Jackson, Yu-Tao Xiang

**Affiliations:** 1 Department of Public Health and Medicinal Administration, & Institute of Translational Medicine, Faculty of Health Sciences, University of Macau, Macao SAR, China.; 2 Centre for Cognitive and Brain Sciences, University of Macau, Macao SAR, China.; 3 Institute of Advanced Studies in Humanities and Social Sciences, University of Macau, Macao SAR, China.; 4 The National Clinical Research Center for Mental Disorders & Beijing Key Laboratory of Mental Disorders, Beijing Anding Hospital & the Advanced Innovation Center for Human Brain Protection, Capital Medical University, Beijing, China.; 5 School of Nursing, Hong Kong Polytechnic University, Hong Kong SAR, China.; 6 School of Public Health, Southeast University, Nanjing, China.; 7 Department of Psychology, University of Macau, Taipa, Macao SAR, China.

## Abstract

There has been no consensus about the best public health strategy for managing COVID-19 due to differences in sociocultural, political and economic contexts between countries. The central government of China has emphasized the importance of maintaining the dynamic zero-COVID policy in combating resurgences of new variants. To optimize the dynamic zero-COVID policy for future COVID-19 outbreaks in China, this article outlines a comprehensive strategy that should be considered.

## Introduction

China's zero-COVID policy has garnered considerable attention globally and stimulated much debate 1. Since the novel coronavirus-19 (COVID-19) first emerged at the end of 2019, a variety of strategies has been undertaken to control the pandemic in different countries. In China, to cope with the spread of the highly transmissible Delta variant, a new strategy entitled the “Dynamic zero-COVID” was adopted in August 2021; this strategy has been also used in response to later transmissible variants such as Omicron 2. At present, there has been no consensus about the best public health strategy for managing COVID-19 due to differences in sociocultural, political and economic contexts between countries. Based on lessons drawn from managing past disease outbreaks, China has adopted a dynamic zero-COVID policy in which restrictive measures are initiated and maintained until there are no documented COVID-19 cases in a particular geographic location.

In recent months, the dynamic zero-COVID policy has been questioned in light of increased transmissibility of new, often milder SARS-CoV-2 variants (e.g., Omicron) and mounting economic and personal costs of implementing the policy. In this policy, strict quarantine and management measures are implemented if necessary, and may increase the risk of various mental health problems. In response, mental health associations and academic societies have organized expert teams and released various guidelines for mental health services in China 3. In the context of a growing vaccination rate, decreasing COVID-19 fatality rate, and significant economic impact of mass lockdowns, suggestions have been made for de-escalation and easing of restrictions 1. In response, during early May 2022, the central government of China emphasized the importance of maintaining the dynamic zero-COVID policy in combating resurgences of new variants. This policy has faced some opposition in Shanghai where the recent COVID-19 outbreak has resulted in over 40,000 confirmed symptomatic cases and 500,000 asymptomatic cases during one month in 2022. Nonetheless, the policy has also been effective in containing clusters of Omicron infections in other large Chinese cities such as Shenzhen. To optimize the dynamic zero-COVID policy for future COVID-19 outbreaks in China, a comprehensive strategy that integrates certain important factors should be considered.

First, potential effects of the dynamic zero-COVID policy on health services should be considered. Unquestionably, the dynamic zero-COVID policy has saved many lives. A recent study 4 estimated that the 2022 Omicron outbreak could cause approximately 1.55 million deaths nationwide if stringent COVID-19 controls were lifted without wider vaccine coverage and access to antiviral therapies. Another study based on the recent Shanghai epidemic data predicted the peak of newly confirmed cases and newly asymptomatic infectious and suggested that earlier implementation of strict control measures would result in an earlier downturn in the epidemic. These findings reinforced resolve in maintaining the dynamic zero-COVID policy 5.

That said, strict lockdowns can have an enormous impact on health services for those with chronic diseases. China-based research 6 has found that routine checkups and follow-up treatments for heart disease, stroke, chronic kidney disease, and cancer patients may be compromised by insufficient medical resources, many of which have been diverted to combat the pandemic. During the recent Omicron wave in Shanghai, strict lockdowns have induced havoc among residents, particularly due to interruptions in regular medical care and more limited treatment resources. For example, patients may be unable to receive dialysis due to strict hospital policies of no admission if a negative polymerase chain reaction (PCR) test for COVID-19 cannot be provided 1. In response, the Shanghai health authority has adopted timely measures (e.g., online booking, registration, specialist consultation, prescription, medical insurance payment and home drug deliveries) to remediate these problems. These strategies have been integrated immediately into the COVID-19 policy so that subsequent Omicron waves or variants can be combatted in other Chinese cities such as Beijing 7.

The influence of COVID policies on economic development should also be considered. For instance, economic damage caused by the recent coronavirus outbreak in Shanghai could be more than 10 times higher than economic effects of the initial outbreak in Wuhan during early 2020, wherein 13 million people were affected and related economic losses were estimated to be 1.7 trillion yuan 8. The Chinese government has made significant efforts to balance the COVID policy with economic growth considerations during the pandemic. Successful implementation of the dynamic zero-COVID policy in certain metropolitan areas of China could also minimize negative effects of variants on the economy. For instance, Shenzhen intervened early in response a local outbreak in early 2022 by introducing a sweeping lockdown and three rounds of city-wide mass testing. After lifting the lockdown one week later, infections had decreased and economic activity resumed at close to normal levels 9.

Recently, the WHO chief questioned the extent to which a zero-COVID policy is sustainable in the face of potentially increased transmissibility of Omicron and other variants. However, at present there is no certainty in how the virus will evolve so the appropriateness of any particular policy for managing the pandemic may only be clear in hindsight. There is no doubt that dynamic surveillance of variations in SARS-CoV-2 and the development of effective antiviral treatments are needed. Consequently, COVID policies may need to be refined in response to potential pandemic effects on health services, chronic diseases, psychological well-being and economic factors. Ideally, flexible, data-guided approaches tailored to suit different sociocultural, economic and political contexts of affected countries and territories as well as changes in the virus itself can be adopted. Based on experiences and lessons learned in response to recent Omicron waves, we believe that China can effectively combat the pandemic with an optimized dynamic COVID-19 policy.
